# The identification of sulfide oxidation as a potential metabolism driving primary production on late Noachian Mars

**DOI:** 10.1038/s41598-020-67815-8

**Published:** 2020-07-02

**Authors:** M. C. Macey, M. Fox-Powell, N. K. Ramkissoon, B. P. Stephens, T. Barton, S. P. Schwenzer, V. K. Pearson, C. R. Cousins, K. Olsson-Francis

**Affiliations:** 1 AstrobiologyOU, Faculty of Science, Technology, Engineering and Mathematics, The Open University, Milton Keynes, UK; 2 School of Earth and Environmental Sciences, University of St Andrews, Irvine Building, St Andrews, UK

**Keywords:** Microbiology, Microbial ecology, Astrobiology, Geochemistry

## Abstract

The transition of the martian climate from the wet Noachian era to the dry Hesperian (4.1–3.0 Gya) likely resulted in saline surface waters that were rich in sulfur species. Terrestrial analogue environments that possess a similar chemistry to these proposed waters can be used to develop an understanding of the diversity of microorganisms that could have persisted on Mars under such conditions. Here, we report on the chemistry and microbial community of the highly reducing sediment of Colour Peak springs, a sulfidic and saline spring system located within the Canadian High Arctic. DNA and cDNA 16S rRNA gene profiling demonstrated that the microbial community was dominated by sulfur oxidising bacteria, suggesting that primary production in the sediment was driven by chemolithoautotrophic sulfur oxidation. It is possible that the sulfur oxidising bacteria also supported the persistence of the additional taxa. Gibbs energy values calculated for the brines, based on the chemistry of Gale crater, suggested that the oxidation of reduced sulfur species was an energetically viable metabolism for life on early Mars.

## Introduction

Sulfurous and saline waters are proposed to have existed on the surface of Mars during the Noachian–Hesperian transition (4.1–3.0 Gya)^1–6^, whereby in the Noachian period, liquid water formed widespread surface features, such as stream beds and sedimentary deposits, and led to the depositions of clay minerals^4^. Many locations on Mars feature rock formations rich in sulfur species, with sulfate and sulfide minerals detected by lander missions^7–11^ and within martian meteorites^12–16^. At the end of the Noachian and into the beginning of the Hesperian, the presence of water declined and saline-rich brines formed, as evidenced by the jarosite at Gale crater^17^. On modern day Mars, water is restricted to the subsurface or—potentially—sub-glacial areas at the poles^4,18,19^.

In sulfur-rich environments on Earth, primary production is typically driven by the oxidation of reduced sulfur species^20–23^. The sulfur biogeochemical cycle involves metabolic activity associated with multiple microbial pathways, and molecular and physiological data indicate that these sulfur oxidation–reduction (redox) reactions are an ancient metabolism^24^. The presence of sulfur species in different redox states on Mars and the detection of suitable electron donors and acceptors (e.g. nitrate and oxygen^25–27^) raises the possibility of whether the sulfur biogeochemical cycle, specifically the oxidation of reduced sulfur species, is plausible on Mars. This is especially important, because Mars offers a wide range of environmental conditions, mostly dominated by basalt-water reactions, with pH varying from alkaline^28,29^ to acidic (in conjunction with volcanic activity)^30^. The availability of water and water activity have been invoked as limiting factors for habitability^31,32^, but predictions suggest a range of water chemistries have existed through martian geological time, from the dilute “groundwater-type”^28,29^ to the highly concentrated brines associated with the modern day recurring slope lineae occurrences^17,33^.

**Figure 1:**
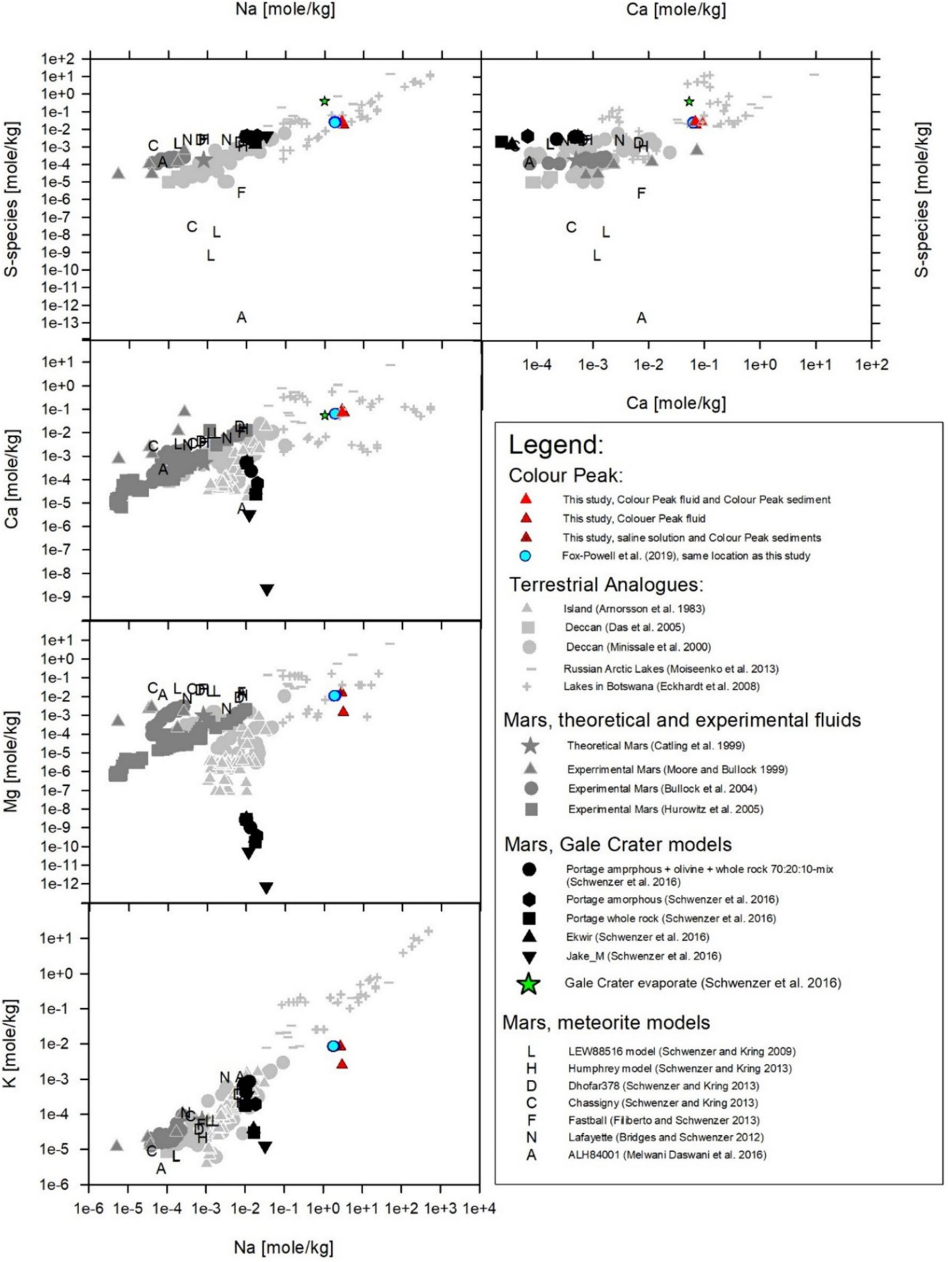
Colour Peak water compared with other terrestrial analogues, including waters from arctic lakes and evaporating lakes in Botswana, and of martian fluids (theoretical, experimental and modelled)^46, 47^.

**Figure 2:**
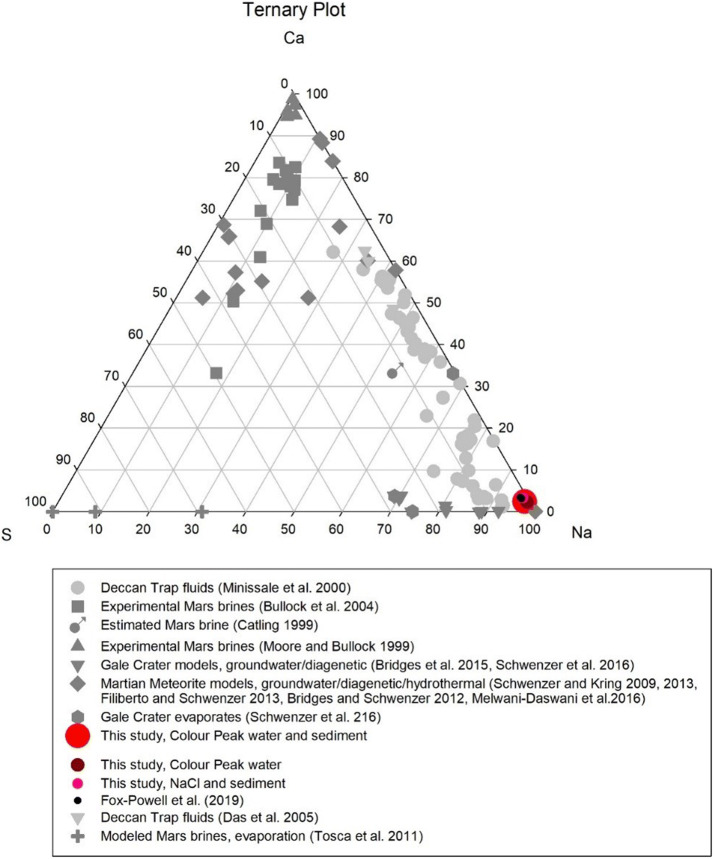
Ternary plot showing the concentrations of Na, Ca and S in waters from the Colour Peak spring and sediment compared to fluids from the Deccan traps^48,49^ and modelled Mars brine chemistries^28,29,50–57^.

Based on the tiered model method of Soare^34^, Axel Heiberg Island in the Canadian High Arctic represents an ideal analogue to study microbial processes within sulfurous aqueous environments similar to those that existed on Mars, e.g., at Gale crater at the Noachian-Hesperian boundary. The brines were sulfur-rich and became concentrated by processes such as freezing or evaporation^29^. Figures 1 and 2 illustrate this point, with sulfate-vein forming fluids at Gale crater being most similar to fluids in saline lakes on Earth and in the Axel Heiberg spring. At the same time, the fluids from clay formation events, and more generally the alteration of basaltic material under high water–rock reactions on Mars, matches fluids from terrestrial geologic settings, such as the Deccan traps and Icelandic springs (see Figs. 1 and 2)^1–5,35–39^.

Axel Heiberg Island lies within the region of continuous permafrost^40,41^ and is host to eight sulfur–rich, highly saline (2–4 M), and perennially cold (0–7 °C) springs^41–43^. Despite an average air temperature of − 15 °C, which decreases to a minimum of − 40 °C in winter, the springs do not freeze^2,3, 36, 44^. The water in the island’s spring systems persists as groundwater 600 m below the surface. The water discharges in areas associated with diapiric uplift^38^, which comprises of gypsum–anhydrite of upper-Mississippian to middle-Pennsylvanian age (224–315 Mya)^13^. The waters are anoxic upon exiting the diapirs and become rapidly oxidised at the surface^2,42^. The springs that have been previously characterised with regards to their chemistry and mineralogy are Lost Hammer (LH), Gypsum Hill (GH) and Colour Peak (CP)^2,38,42,43^. These studies have indicated that the mineralogy of deposits at these spring sites are predominantly calcite (CaCO_3_), halite (NaCl), thenardite (Na_2_SO_4_), mirabilite (Na_2_SO_4_·10H_2_O), gypsum (CaSO_4_·2H_2_O) and elemental sulfur (S°)^38,42,43^, but variations exist between springs as a result of differences in their fluid geochemistry. For example, LH fluids have higher concentrations of sodium than CP fluids, but lower concentrations of sulfide (0.14 mM) and calcium (24.43 mM) than CP (1.8 mM and 33.23 mM respectively)^43^. The sediments of the Axel Heiberg springs are typically highly reducing, with some sediments containing both anoxic and microaerophilic zones^38^.

**Figure 3:**
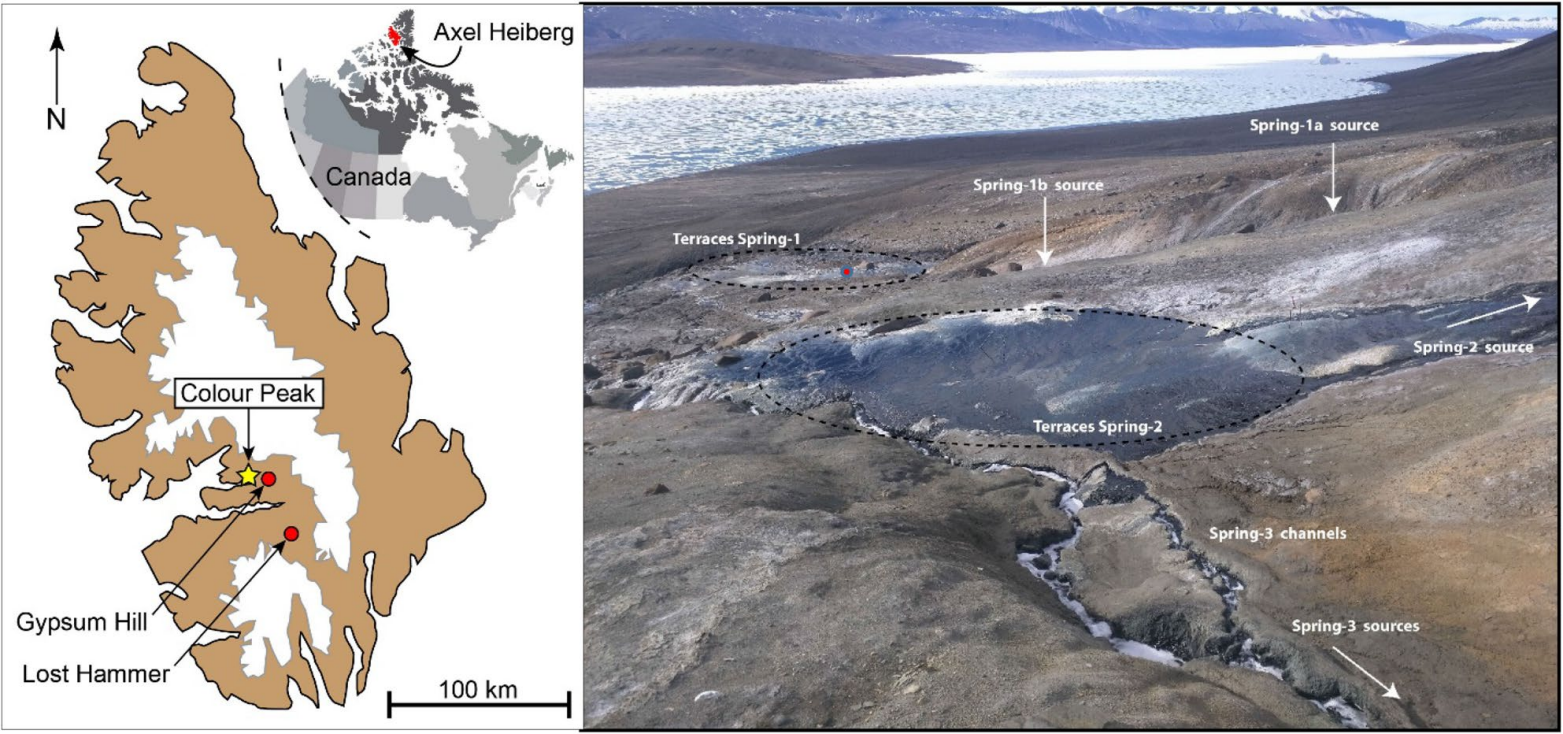
(**A**) Map of Axel Heiberg Island and (**B**) photograph of the Colour Peak (CP) site illustrating the location of CP springs and their sources. (**A)** Map of Axel Heiberg Island (brown), and icecaps (white) produced by modifying an image from Google Maps (Map Data @2020 Google) using Illustrator Creative Cloud version 21.0.2. (**B**) Photographs of the CP springs and precipitates along a CP spring channel with annotated spring and channel systems. The site where the sediment was collected is marked with a red dot.

The CP spring system consists of a series of springs that discharge into deep gullies located near the base of the south–facing slope of CP, as shown in Fig. 3^43^. The CP spring waters possess a broadly similar composition and pH (7.3–7.9^43^) to that of a thermodynamically–modelled martian evaporitic fluid (based on the chemistry of Gale crater, Figs. 1 and 2). The CP spring system is therefore a recognised Mars analogue site^3, 45^ that possesses a chemistry similar to that proposed for the late Noachian era. However, despite several studies investigating the springs on Axel Heiberg Island, specific microbiological studies of the CP spring system have been less forthcoming. The resident microbial community has previously only been characterised through the creation and sequencing of clone libraries (174 clones for bacterial diversity and 164 for archaeal diversity) and no strains have been isolated from this site^45^.

Conversely, LH and GH have been studied using 16S rRNA gene analysis and a multiple amplification enabled metagenome^44,45,58–62^. At higher taxonomic levels, many of the previous DNA-based studies have shown microbial communities dominated by Gammaproteobacteria, specifically those genera that are associated with sulfur oxidation^45,60–62^, a factor not detected in the cDNA profiles of the LH springs^58,59^. However, the preservation of environmental DNA under high salinity and cold temperatures^63–66^ limits the extent to which the bacterial community within the sediment can be characterised using this approach, since the 16S rRNA gene profile also captures sequences from dead cells. This necessitates the analysis of cDNA produced from RNA extracted from the sediment.

This paper presents the first in-depth characterisation of the viable microbial community at CP springs, determined through DNA and RNA extraction from a CP sediment core. cDNA was produced from the RNA and 16S rRNA amplicons were sequenced using current generation sequencing platforms. Several halophilic bacteria were isolated, representing the first cultivation-dependent characterisation of this site. The viability of metabolisms under thermochemically-modelled martian fluids was also estimated using Gibbs energy (formally called Gibbs free energy) equations to further investigate the habitability of the waters on Mars from the late Noachian.

## Results

### Geochemical characterisation

Inductively Coupled Plasma–Optical Emission Spectroscopy (ICP–OES) was used to identity the bioavailable elements in: (1) water from CP; (2) CP water that had CP sediment resuspended in it for seven days; (3) 17% NaCl solution that had CP sediment resuspended in it for seven days, and (4) 17% NaCl solution analysed as a control (Table 1). The CP fluids contained high amounts of sodium (69,600 mg kg^−1^), calcium (2,850 mg kg^−1^), sulfur (563 mg kg^−1^) and potassium (97 mg kg^−1^). Interaction with the CP sediment increased the concentrations of potassium (threefold), magnesium (tenfold), strontium (ninefold) and sulfur (1.5-fold). The concentrations of sodium and calcium did not alter from contact with the sediment. The high concentrations of sulfur present in the CP waters and sediment is congruent with the prior detection of sulfur oxidising bacteria within the CP springs^45^, as this represents a highly abundant and bioavailable electron donor and would be expected to impact on the resident microbial community.

**Table 1:** Major and minor elements (mg/kg) in the Colour Peak water and liberated from the Colour Peak sediment.

	Minimum detection limits (mg/kg)	Colour peak water	Colour Peak water after resuspension of sediment	NaCl solution	NaCl solution after resuspension of sediment
Al	0.014	0.23 (0.04)	0.68 (0.02)	0.19 (0.02)	0.64 (0.03)
Ca	0.002	2,850 (1,020)	2,760 (1,100)	< 0.002 (0)	3,740 (1,240)
K	0.015	97.50 (2.90)	338 (5.00)	< 0.015 (0)	325 (0)
Mg	0.001	35.0 (1.94)	386 (2.11)	< 0.001 (0)	405 (0)
Rb	0.002	1.25 (0.08)	3.08 (0)	< 0.002 (0)	2.33 (0)
S	0.010	563 (2.90)	900 (48.8)	2.92 (2.01)	843 (0.17)
Si	0.002	1.35 (0.55)	2.86 (0.15)	2.71 (0.25)	8.38 (0.14)
Sn	0.015	0.25 (0.08)	0.33 (0.08)	< 0.015 (0)	0.67 (0.08)
Sr	0.001	11.8 (0.46)	90.1 (0.72)	< 0.001 (0)	92.4 (0)
Na	0.043	69,600 (586)	63,100 (381.24)	74,000 (689)	63,000 (433)

Note especially the high concentrations of sulfur, sodium and calcium and the enhanced concentration of sulfur following sediment resuspension.

Standard deviation is in brackets.

### Gibbs energy calculations

The Gibbs energy (*ΔG*) of sulfide oxidation was calculated to assess the energetic feasibility of this reaction within thermochemically-modelled martian brines (with chemistries similar to CP waters)^57^. The fluid chemistries used for the Gibbs energy calculations were modelled using the concentration of oxygen shown to be viable in the martian near-surface^67^ and the concentration of nitrate detected in ancient mudstones by the Mars Curiosity rover (e.g., the lower and upper limits of nitrate were 70 and 1,200 ppm respectively^26^) (Supplementary Table 1). *ΔG* values indicated that aerobic sulfide oxidation was viable, yielding 2.36 × 10^–4^ kJ kg^−1^_(fluid)_ in the fluids modelled with both 70 and 1,200 ppm of nitrate. Denitrification-fuelled sulfide oxidation was also shown to be viable, yielding 2.21 × 10^–5^ kJ kg^-1^_(fluid)_ in the modelled fluid chemistries with 70 and 1,200 ppm of nitrate. In terms of cell biomass, this translates to 2.97 × 10^7^ cells kg^−1^_(fluid)_ supported by aerobic sulfide oxidation and 2.78 × 10^6^ cells kg^−1^_(fluid)_ supported by anaerobic sulfide oxidation. Other potential metabolisms were viable (Supplementary Table 1), but none were more energy yielding than sulfide oxidation.

### Microbial community within the colour peak sediment

DNA and RNA (converted to cDNA) were extracted from replicate sediment samples collected from CP and sequenced using the Ion Torrent PGM platform. 113,278 16S rRNA gene sequences were obtained from the DNA extractions, post-quality control (Supplementary Table 2). Sequences were evenly distributed between the three samples examined (35,201 Sample 1 (S1); 37,206 at Sample 2 (S2); and 40,871 at Sample 3 (S3). cDNA produced from RNA was extracted from the same three sediment samples, pooled, and 41,955 16S rRNA sequences obtained post-quality control.

**Figure 4:**
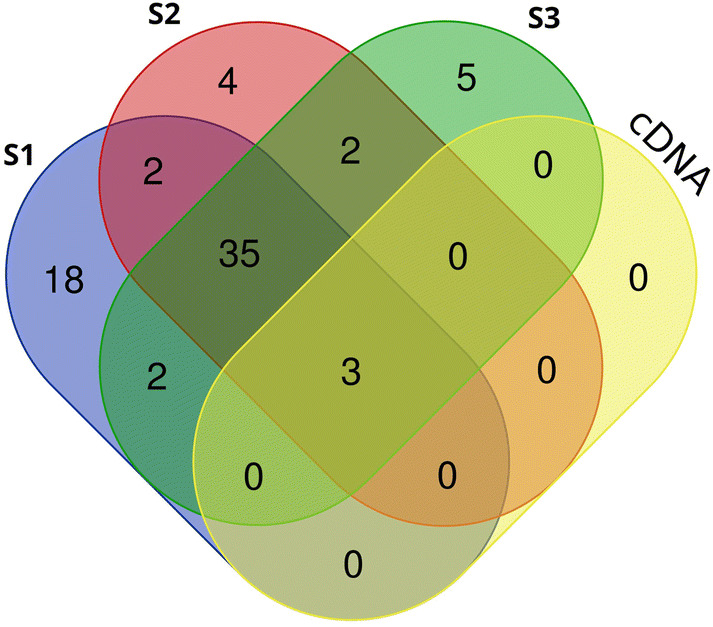
Shared diversity at the genus level between the 16S rRNA gene and 16S rRNA profiles of the Colour Peak sediment. All genera present at greater than 100 reads in the 16S rRNA gene and 16S profiles were compared to identify shared and unique genera between the three replicate sediment samples and the RNA profile of Colour Peak sediment.

Alpha diversity metrics were applied to both data sets to assess the community diversity. The number of operational taxonomic units (OTUs, sequences with > 97% sequence identity) in the 16S rRNA gene profile ranged from between 347 and 401 (S1, 401 OTUs; S2, 347 OTUs; S3, 376 OTUs). 94–98% of the OTUs present in the 16S rRNA gene profiles were common to each sample (Fig. 4). Whilst there were over 300 OTUs detected in the DNA profiles, only 33 were detected in the cDNA data. Diversity indices (Faith pd, Shannon, Simpson and Simpson evenness) confirmed that the diversity in the 16S rRNA gene profiles was greater than that of the 16S rRNA profile of the cDNA (Supplementary Table 3). Beta diversity metrics (Euclidian Distance, Dice measure, Chebyshev distance) were applied to identify variation in community structure between the DNA and cDNA profiles and showed that there was less variation within the DNA profiles compared to the cDNA profile (Supplementary Tables 4, 5 and 6).

**Figure 5:**
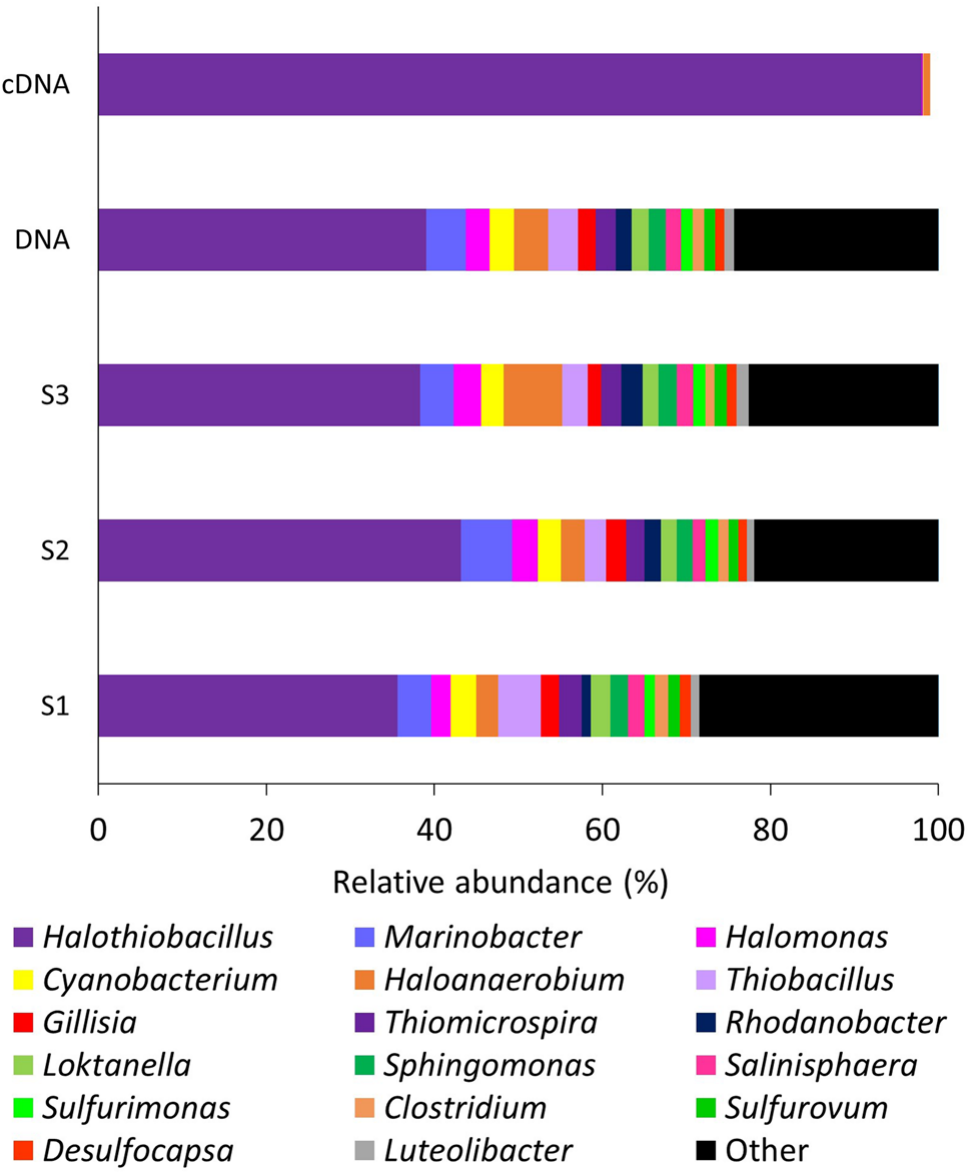
16S rRNA gene and 16S rRNA community profiles of Colour Peak (CP) sediment. Sequences were revealed by amplicon sequencing of 16S rRNA gene and 16S rRNA amplicons retrieved by PCR from DNA and RNA extracted from three replicate sediment samples (S1, S2, S3) collected from CP and the pooled 16S rRNA profile of the CP sediment. All genera pictured are present at > 1% relative abundance. DNA refers to an averaged community profile of the three replicate CP 16S rRNA gene profiles.

Taxonomical assignment demonstrated that the majority of the sequences in the DNA profiles belonged to the phylum Proteobacteria (Fig. 5), which represented between 74% (S1) and 78% (S2) of the relative abundance. Sequences that were assigned to the phylum Proteobacteria, were mainly identified at Class level as Gammaproteobacteria (74–80%). This was followed by the Alphaproteobacteria (8–10%), Bacteroidetes (8%), Firmicutes (6–9%), Betaproteobacteria (4–6%), Epsilonbacteraetota (2–3%) and Cyanobacteria (3%). *Halothiobacillus* was the most abundant genus (on average 40% of the relative abundance of the total community profile). Other gammaproteobacterial genera associated with sulfur oxidation were also present (*Thiobacillus*, *Thiomicrosospira*, *Halomonas*, *Marinobacter* and *Salinisphaera* (> 1% of the total relevant abundance)). With regards to Archaea, the amplicons produced with the universal primers contained no archaeal sequences and the screening of the cDNA and DNA with archaeal specific primers did not produce a successful amplicon. Whilst the lack of detection of Archaeal signatures is unexpected, halophilic bacteria have previously been shown to be capable of outcompeting halophilic archaea under moderately saline (20%) and colder conditions^68^, which may have resulted in either the competitive exclusion of the archaea or a reduction in their abundance within the site.

100% of the cDNA profile was comprised of genera that were also identified in the 16S rRNA gene profiles (Table 2) and was dominated by Proteobacteria (99%), specifically the Gammaproteobacteria (99%). The dominant genus of the 16S rRNA profile was *Halothiobacillus*, representing 98% of the relative abundance in the cDNA profiles. Other genera present at a read number greater than 100 were *Halomonas* and *Halanaerobium*.

**Table 2:** Read numbers for the most abundant genera in the Colour Peak (CP) 16S rRNA gene and 16S rRNA profiles obtained from replicate sediment samples.

Genus	CP S1	CP S2	CP S3	CP cDNA
*Halothiobacillus*	12,472	15,102	13,392	34,300
*Thiobacillus*	1,376	2,133	1,412	3
*Marinobacter*	820	1,063	1,138	0
Cyanobacteria	1,066	983	951	25
*Thiomicrospira*	908	975	2,435	0
*Halanaerobium*	1785	892	1,052	243
*Halomonas*	758	845	575	110
*Loktanella*	942	769	841	0
*Salegentibacter*	393	677	886	0
*Sphingopyxis*	798	659	655	0

### Isolation and identification of microbial isolates

19 isolates representing 11 genera were isolated from the sediment (Supplementary Table 7). All of the isolates belonged to the genera *Halomonas, Psychrobacter, Marinobacter, Loktanella, Salegentibacter, Sphingopyxis, Sporosarcina, Variovorax, Acidovorax* and *Nevskia* (> 98% identity to known NCBI sequences). Three strains that were isolated from the CP sediment, *Psychrobacter* (CP4 and CP5) and *Sporosarcina* (CP16), belong to genera that were not present in the sequencing profiles. This is perhaps a result of the enrichment process which has increased the abundance of strains from genera that were present in the natural community at sufficiently low abundances to preclude their detection in the 16S rRNA gene amplicons.

## Discussion

In this study, high-throughput sequencing was utilised to study the microbial community of CP, a saline and sulfate-rich spring system located in the Canadian High Arctic, which is chemically similar to waters proposed to have been present on the surface of Mars during the late Noachian^1–5^. 16S rRNA gene data demonstrated that the sediment was dominated by members of gammaproteobacterial genera that are comprised solely of obligate chemolithoautotrophic sulfur oxidisers^69–71^, specifically *Halothiobacillus* (35–43% relative abundance) and *Thiomicrosopsira* (2–3% relative abundance), or contained species that were capable of complete or partial sulfur oxidation, for example *Marinobacter* (4–6%) and *Halomonas* (2–3%)^72,73^. Sequences belonging to *Sulfurovum* and *Sulfurospirillum* (members of the Epsilonbacteraetota Phylum) together represented 3% of the community profile. The dominance of diversity associated with sulfur-related metabolisms is presumably due to the high concentrations of bioavailable sulfur detected within the sediment and waters of the CP spring system. The genera associated with sulfur oxidation that are most abundant in the CP sediment are most commonly associated with the oxidation of sulfide to sulfite by sulfite reductase and subsequent transformation of the sulfite to thiosulfate by thiosulfate sulfurtransferase. The thiosulfate is then oxidised to sulfate via the enzymes of the Sox pathway^69–71^. Species of *Thiobacillus* that were also detected in the CP sediment have also been shown to complete sulfur oxidation via the oxidation of sulfite to sulfate by a sulfite dehydrogenase and via the reverse dissimilatory sulfate reduction pathway^71^. Sulfate is the end product of these sulfur oxidation pathways, but with the accumulation of thiosulfate, sulfur and sulfite as metabolic intermediates shown to occur within the diversity detected^71^.

The results from this study are consistent with previous 16S rRNA gene profiles of the LH, GH and CP spring sediments that showed a dominance of Gammaproteobacterial genera associated with sulfur oxidation^45,58,60,62^. However, previous 16S rRNA profiling of the LH springs showed a dominance of either Chloroflexi and Alphaproteobacteria and a minimal presence of Gammaproteobacteria^40^ or showed abundant Gammaproteobacteria but this was diversity not associated with sulfur oxidation^38^. This study, however, showed that the CP sediment was dominated by Gammaproteobacteria, specifically the genus *Halothiobacillus* (98% relative abundance). This result indicates that chemolithoautotrophic sulfur oxidation is an active process within the CP sediment.

As well as water chemistry, another key environmental parameter of this site that is analogous to Mars is the low temperature, which models have shown exist at atmospheric pressures between 100 mbar and 4 bar that can be constrained through the investigation of carbonates and sulfates^18,74,75^. Climate models indicate that, during the Noachian, Mars would have experienced seasonal temperature variations around the freezing point of water at low latitudes. This would have allowed surface water features, such as rivers and lakes, to form and be sustained^18,76^, but on modern day Mars water reservoirs are expected to be found in the subsurface^77–80^. This is consistent with the temperatures observed in CP, with air temperatures consistently below freezing. This is reflected in the fact that the majority of the isolates (e.g. *Marinobacter, Halomonas, Loktanella* and *Psychrobacter*) obtained in this study are > 98% homologous to isolates previously detected in the Arctic or other cryoenvironments, including GH and LH springs^44,45,81–84^. Further, the 16S rRNA profile, 16S rRNA gene sequence data and isolates gained from this study reinforce the similarities in community composition between the separate sites on Axel Heiberg island identified in previous studies^59,60,62^. This could be explained by the sites having a common, or related, water source^42^, but determining the relative roles of stochastic and deterministic factors that might control community composition in the sediments would require a larger and more rigorous sampling effort^85^. The survival and growth of these organisms within the CP sediment suggests that they are suitable candidates for studies simulating the martian chemical environment.

Within the CP sediment, functional guilds associated with both obligate anaerobism (e.g., fermentative metabolisms) and aerobism (e.g., sulfur-oxidising metabolisms) were identified in the 16S rRNA profile. The continued viability of obligate anaerobes within the CP sediment proves that conditions enabling their survival exist, possibly within anaerobic microenvironments within the heterogeneous sediment^38^. Based on the chemistry and the community profiling of the CP spring sediment, the comparison with sulfur-rich Mars raises the potential for the oxidation of reduced sulfur species to be an energy yielding metabolism that could fuel primary production within ancient martian environments, or even in modern subsurface environments. Further, sulfide-bearing minerals, including pyrite and pyrrhotite, have been detected in martian meteorites and via in-situ measurements from the martian surface^14,15^, which could be used as electron donors for this metabolism.

On Earth, the oxidation of reduced sulfur compounds by sulfur oxidising bacteria can be coupled to either oxygen (under aerobic conditions) or nitrate (under microaerophilic or anaerobic conditions) as electron acceptors. For example, this occurs in marine sediments and hydrothermal vents, where there is limited light and a gradient in the availability of oxygen and nitrate^86,87^, and in artificial environments, such as wastewater treatment plants, which have higher concentrations of nitrate^88,89^. On Mars, the oxidation of sulfur species could be coupled to these electron acceptors in a similar manner^90^. For example, oxygen has been detected in the martian atmosphere by the Curiosity rover^25^, with thermodynamic models indicating that subsurface environments on Mars could possess sufficient O_2_ to allow for aerobic metabolisms to be viable^67^. The Curiosity rover has also detected nitrates in ancient mudstones analysed at Gale crater at 70–1,100 ppm^26^. These values were used in the Gibbs energy calculations presented here and showed that, in the presence of modelled Mars-relevant brines, both aerobic and anaerobic sulfide oxidation are thermodynamically viable in a martian chemical environment.

Using the concentrations of oxygen shown to be thermodynamically feasible in brines under near surface conditions^67^, aerobic sulfide oxidation was shown to support a greater number of cells than denitrification-fuelled sulfide oxidation (2.97 × 10^7^ cells kg^−1^_(fluid)_ and 2.78 × 10^6^ cells^−1^_(fluid)_, respectively). Phototrophic sulfur oxidation may have also been a plausible metabolism in the late Noachian. However, as phototrophy is dependent on light, it is restricted to the surface and not viable in the sub-surface sediment environments considered here. Denitrification-fuelled sulfide oxidation was shown to be viable with both the higher and lower concentrations of nitrate detected in the mudstone from Gale crater, suggesting that this metabolism would have been feasible within a broad range of potential environments on Mars during the late Noachian.

If sulfur-oxidising microorganisms existed on Noachian Mars, evidence of their activity might be preserved in Noachian-aged martian sediments. The entombment of microbial lipids within iron sulfates and iron oxides might be used to identify the presence of life, however there is ambiguity with regards to the identity and metabolisms of the microbes associated with the lipids^91^. Biosignatures more specific to sulfur oxidising bacteria include the enhanced formation and altered composition of specific biominerals^92,93^, e.g., the enhanced production of gypsum^38^ or the substitution of calcium by barium in gypsum^94^. Specific sulfur oxidising bacteria also possess unique budding and filamentous cell morphologies that could be preserved in specific environmental systems^95^. However, the concentration of sulfur within a system cannot be used as a reliable biosignature, since different sulfur oxidising bacteria are known to either accumulate or enhance the removal of extracellular sulfur^92,96–98^. Sulfur isotopic fractionation patterns, however, could be utilised since the oxidation of reduced sulfur compounds by sulfur oxidising bacteria has recently been shown to enrich its oxidation products with ^34^S^99^. Although variable sulfur isotopic compositions have been observed between sediments at Gale crater on Mars, this does not allow the conclusion for a biological origin because it is at present not possible to discount all non-biological reasons for these differences^16^.

In addition to autotrophic sulfur oxidisers, the cDNA profile of the CP sediment included strains of fermentative bacteria and *Halomonas*, a genus that includes heterotrophic strains^100^ that require an exogenous source of organic carbon. Carbon has been detected on the surface of Mars and in martian meteorites^101^. Sutter^101^ postulated that < 1% of the carbon detected at Gale crater would support the biomass requirements for 1 × 10^5^ cells g^−1^ sediment^101^. In addition, if sulfur biogeochemical cycling occurred on Mars, the organic carbon could be supplied via the secretion and necrophagy of sulfur oxidising bacteria^102^. The role of sulfur oxidising bacteria as primary producers within environments raises the issue of whether *Halothiobacillus* could therefore be considered a keystone species or sulfur oxidation a keystone function in the CP sediment^85^, enabling the viability of additional metabolisms. If so, it could be extrapolated that this could also occur under proposed martian conditions (Supplementary Fig. 1). Syntrophy and co-cultivation have been shown to be highly influential to the persistence of microbial populations^103–105^. Therefore, in a community-dependent context, a greater diversity of metabolisms might be viable, which could have profound implications for the formation and preservation of biosignatures under martian chemical conditions.

## Conclusion

Constraining the parameters concerning biosignature formation in the former and potentially extant waters of Mars requires the identification of organisms capable of surviving in sites that represent appropriate analogue environments. The sulfidic, sulfurous and saline conditions of the CP spring system on Axel Heiberg Island represent such an environment, as an analogue for waters on the surface of Mars during the Noachian–Hesperian transition. This study shows that the microbial community within the CP sediment was dominated by bacteria associated with the oxidation of reduced sulfur species. Based on thermochemical models for the sulfur-rich brines of the Noachian–Hesperian period, conditions could have been thermodynamically viable for similar biotic sulfur oxidation to occur. The potential role of chemolithoautotrophic sulfur oxidation as a keystone function that drives primary production and helps to maintain diversity in terrestrial environments has implications for our understanding of the habitability of martian environments by non-chemolithoautotrophic metabolisms and the subsequent impacts on biosignature formation. The relationship and dependencies between metabolisms require further exploration under controlled laboratory conditions, with simulation studies presenting an appropriate method for achieving this future goal.

## Methods

### Site sampling and description

Samples were collected during the summer field season in 2017. Sediment samples were aseptically collected from a sediment-rich pool (79.381359°, − 91.272664°) for molecular analysis and culturing and were stored at ambient arctic temperatures whilst in the field. All tools used to collect the samples were cleaned with 95% ethanol and rinsed with autoclaved ddH_2_O between sampling. Approximately 150 g of sediment was collected and stored in sterile 50 ml tubes (for molecular work) and 125 ml Nalgene bottles (for culturing). The samples were shipped to the UK chilled (4 °C) and on return to the laboratory were stored at – 80 °C (for molecular analysis) and 4 °C (for culturing). Temperature, pH and dissolved oxygen (DO) concentrations were measured in situ using a Mettler Toledo FiveGo probe.

### Nucleic acid extraction

The extraction process was performed in a clean hood (PURAIR, Air Science) used exclusively for low biomass samples. Prior to use, the hood was sterilized with 2% chemgene and RNaseZap (ThermoFisher) and then UV sterilized for 72 h. The extractions were performed using the XS buffer extraction technique^106^. All reagents, except the potassium ethyl xanthogenate, were UV sterilized and after preparation the XS buffer was filter–sterilized through a 0.22 µm filter. For each stage of the extraction process, nuclease–free water (Sigma) was introduced as an additional negative control. All controls were processed in parallel with the samples and used as negative extraction controls in the PCRs.

10 g from each sediment sample was suspended in 30 ml of PCR grade molecular water (Sigma). The sediment was vortexed for 20 min prior to centrifugation at 1,000×*g*. After 5 min, the supernatant was removed and centrifuged for a further 5 min at 4,700×*g* through a 15 ml 30 kDa UV sterilised filter (Merck). The tube was emptied, the filters were washed with 400 µL of 1 M pH 8 Tris HCL and then 400 µl of XS buffer was added to the wash solution^106^. DNA was extracted from the filtered samples using the XS buffer DNA extraction technique, with the addition of a freeze–thaw step, with the samples being stored at – 80 °C for 30 min after 30 min of incubation at 65 °C^106^. Nucleic acids were precipitated with one volume of ethanol and 4 µl of GlycoBlue Coprecipitant (ThermoFisher), as per manufacturer’s instructions. The supernatant was discarded and the pellet was washed in 200 µl of 70% ice cold ethanol and air dried as in Green et al.^107^.

To remove excess salts, the nucleic acids were re-suspended in 1.5 mL of PCR grade molecular water and then centrifuged at 12,000×*g* for five mins through a 500 µl volume UV sterilised 30 kDA filter (Merck). The filter was inverted, transferred to a new tube, and centrifuged as described previously. The eluted volume was adjusted to a final volume of 40 µL. DNA was quantified using 1 μL with the high sensitivity DNA assay for Qubit fluorometric quantitation (ThermoFisher). Ten microlitres of the nucleic acid suspension was stored for DNA analysis whilst the remaining volume of nucleic acids extracted from each sample was pooled prior to being treated with DNase using the TURBO DNA-free™ Kit (Thermofisher) according to manufacturer’s instructions. Samples were pooled to ensure sufficient yield for successful reverse transcription. To prepare cDNA via reverse transcription, the PCR BIOSYSTEMS qPCRBIO cDNA Synthesis Kit was used according to manufacturer’s instructions.

### PCR amplification and Ion torrent sequencing

Both DNA and cDNA extracts were PCR amplified using a set of primers (515F-806R) specific to the V4 hypervariable region of the bacterial 16 s rRNA gene^108^. The PCR reaction mixture contained (per 25 µL): 1 × PCRBIO Ultra Polymerase red mix (PCR BIOSYSTEMS, United Kingdom), 0.4 μM forward primer and 0.4 μM reverse primer. The PCR conditions were an initial denaturation at 95 °C for 5 min, followed by 30 cycles of: denaturing 30 s at 95 °C, annealing at 1 min 56 °C, elongation at 1 min 72 °C and final elongation for 5 min at 72 °C. PCR products were precipitated as previously decribed^107^, and re-suspended in 20 µl of molecular grade water. Purified PCR products were quantified using Qubit fluorometric quantitation (ThermoFisher) and sequenced using the Ion Torrent PGM platform by the company Molecular Research LP (Texas, USA).

### Bioinformatics analysis

The raw sequencing data was processed using the QIIME2 pipeline^109^. The amplicons were demultiplexed and primers and barcodes removed from all reads. The DADA2 noise removal algorithm was used to remove all chimeric sequences, sequences above 270 bp and the first 15 bp of all sequences. Sequences were then clustered into amplicon sequences variants (ASVs) using the DADA2 algorithm. Phylogeny was assigned to the amplicon sequence variants using Scikit–learn classifier, which compared the ASVs against the Greengenes database^110,111^ with a confidence threshold of *p* = 0.7. The ASVs were aligned using MAFFT^112^ and a rooted tree produced. All of the amplicons were normalised by rarefaction to 35,000 reads and alpha and beta diversity metrics calculated from the normalised data using QIIME2^109^.

### Isolation and identification of bacterial isolates

Microorganisms were isolated from the sample site using a range of media (Supplementary Table 8). The inoculum was prepared by adding 5 g of sediment to 5 ml of 2 M NaCl solution. A 1% inoculum was used to inoculate a series of dilutions (10^–2^–10^–6^), which were incubated at 4–22 °C, for 7–40 days. For isolation, the cultures were plated onto solid media (1.5% agarose) or/and semi-solid media. The individual cells were identified using sequencing of the 16S rRNA gene, using the primers and protocol described previously^113^. Purified PCR products were diluted to 1 ng/μL per 100 bp sequence length and sequenced using Sanger sequencing by MWG Eurofins (Germany). Chromatograms of sequences were analysed using Bioedit (7.0.5)^114,115^ to assess sequence quality. 16S rRNA gene sequences were analysed using the SILVA alignment, classification and tree service to identify the species to which these strains were most closely related^116^.

### Analysis of bioavailable elements in the Colour Peak sediment

Major and trace elements in the CP waters and the bioavailable elements in the sediment were measured by Inductively Couple Plasma-Optical Emission Spectroscopy (ICP–OES) using an Agilent 5110 at the Open University. 5 g aliquots of each sediment were mixed in either 5 ml of sterilised 17% NaCl solution (to simulate the salinity of the CP spring water^43^) or in CP spring water. The samples were incubated at 7 °C for 7 days prior to analysis. For controls, the 17% NaCl solution was analysed in parallel. The accuracy of results was estimated using a 28 component multi-element standard solution for ICP (Fisher Chemical MS102050). The specified wavelength for each element (e.g., Ca 317.933 nm) was selected for repeatability and performance. Check standards for ppm (0.5, 1, 2.5) and ppb (10, 100, 250, 500), blanks and drifts checks were all run to ensure quality control and repeatability of data. Minimum detection limits for individual elements can be found in Table 1 and were derived from three times the standard deviation of the blanks.

### Gibbs energy calculations

Gibbs energy calculations were conducted to determine the feasibility of potential metabolic reactions using Eq. (1)^117^:

1 $$\Delta G = \Delta G^{ \circ } + RT\ln \,\,\,Q$$

where Δ*G* is the Gibbs energy of the reaction, Δ*G*° is the Gibbs energy of the reaction under standard conditions, *R* is the universal gas constant, *T* is the temperature in Kelvin and *Q* is the activity quotient. Activities were determined using the program Spec8 (Geochemist Workbench) and fugacity of atmospheric gases^25,118^. Δ*G*° values were determined using the online SUPCRT programme GEOPIG, which uses the slop07 database. Δ*G* can then be multiplied by the concentration of the limiting reactant (considering the stoichiometry of the reaction) to determine the potential energy available per kg of fluid. To contextualise this, cell densities were estimated using the amount of adenosine triphosphate (ATP) that could be generated from the available energy^119^, where 41.8 kJ is required to make 1 mol of ATP. It was assumed 10% of the available energy would be used to generate new cells^119,120^, 0.02 mol of ATP is required to produce 1 g of biomass and one cell has a mass of 9.50E−13 g. These calculations used brine chemistries determined from thermochemical modelling reported in Bridges and Schwenzer^57^. Concentrations of oxygen modelled as thermodynamically viable within the modern martian near-surface detected in the martian atmosphere and the upper and lower values of nitrates detected in ancient martian sediments by the Curiosity rover were used in these calculations^25,26,67^.

## Supplementary information

Supplementary information.

## Data Availability

Amplicon sequence data generated in this study were deposited to sequence read archives (SRA) under project number PRJNA558950, and Sanger sequence data were deposited to NCBI GenBank under accession numbers MN326776 to MN326790.
