# Supplementary material for: The identification of sulfide oxidation as a potential metabolism driving primary production on late Noachian Mars
Source: Sci Rep. 2020 Jul 2;10:10941. doi: 10.1038/s41598-020-67815-8 (PMC7331718; doi:10.1038/s41598-020-67815-8)
Supplement: Supplementary file 1 — Supplementary information. [file 41598_2020_67815_MOESM1_ESM.docx]

The identification of sulfide oxidation as potential metabolism driving primary production on late Noachian Mars

Macey, M. C. ^1*^, Fox–Powell M. ^1,2^, Ramkissoon, N. K. ^1^, Stephens B. P.^1^, Barton T ^1^., Schwenzer, S. P. ^1^, Pearson, V. K ^1^, Cousins Claire R. ^2^, Olsson–Francis, K.^1^

^1^ AstrobiologyOU, Faculty of Science, Technology, Engineering and Mathematics, The Open University, Milton Keynes, United Kingdom.

^2^ School of Earth and Environmental Sciences, University of St Andrews, Irvine Building, St

Andrews, United Kingdom.

[*michael.macey@open.ac.uk](mailto:*michael.macey@open.ac.uk)

| **Supplementary Table 1. Gibbs free energy calculations of energy yield and estimated number of cells produced from specific metabolisms in a modelled martian fluid chemistry** | | | | | |
| --- | --- | --- | --- | --- | --- |
| **Metabolism** | **Pathway** | **Nitrate concentration of modelled martian fluid chemistry** | | | |
|  |  | **High (1100 ppm)** | | **Low (260 ppm)** | |
|  |  | **Energy available in a kg of fluid (kJ)** | **Number of cells in a kg of fluid** | **Energy available in a kg of fluid (kJ)** | **Number of cells in a kg of fluid** |
| Sulfide oxidation | H_2_S + 2O_2_ = SO_4_^2–^ + 2H^+^ | 2.36E-04 | 2.97E+07 | 2.36E-04 | 2.97E+07 |
| Anaerobic sulfide oxidation | 5HS^–^ + 8NO_3_ + 3H^+^ =5SO_4_^2-^ + 4N_2_ + 4H_2_O | 2.21E-05 | 2.78E+06 | 2.21E-05 | 2.78E+06 |
| Anaerobic iron oxidation | 8Fe^2+^ + NO_3_^–^ + 10H^+^ = 8Fe^3+^ + NH_4_^+^ + 3H_2_O | 2.16E-10 | 2.71E+01 | 2.16E-10 | 2.71E+01 |
| Iron oxidation | 4Fe^2+^ + O_2_ + 4H^+^ = 4Fe^3+^ +2H_2_O | -1.03E-10 | NA | -1.03E-10 | NA |
| Denitrification | NO_3_ + 10H^+^ = N_2_ + 3H_2_O | 1.37E-06 | 1.73E+05 | 1.37E-06 | 1.73E+05 |
| Sulfate reduction | SO_4_^2–^ + H^+^ + 4H_2_ =H_2_S +4H_2_O | 5.14E-07 | 6.47E+04 | 5.14E-07 | 6.47E+04 |

| **Supplementary Table 2. Number of reads from the 16S rRNA and 16S rRNA gene amplicons produced from Colour Peak sediment samples at each stage of processing** | | | |
| --- | --- | --- | --- |
| **Environment** | **Number of reads** | | |
|  | **Raw sequence reads** | **Post quality control** | **Post rarefaction** |
| CP S1 | 139094 | 35201 | 35000 |
| CP S2 | 162091 | 37206 |  |
| CP S3 | 135077 | 40871 |  |
| CP cDNA | 127092 | 41955 |  |

| **Supplementary Table 3. Alpha diversity statistics for the 16S rRNA and 16S rRNA gene profiles of the Colour Peak sediment samples rarefied to 35000 reads** | | | | | | |
| --- | --- | --- | --- | --- | --- | --- |
| **Environmental sample** | **Sample** | **Observed OTUs** | **faith pd** | **Shannon** | **Simpson Eveness** | **Simpson** |
| CP DNA | S1 | 401 | 32.8 | 7.1 | 0.1 | 0.9 |
| CP DNA | S2 | 347 | 28.5 | 6.5 | 0.1 | 0.9 |
| CP DNA | S3 | 376 | 30.0 | 6.9 | 0.1 | 0.9 |
| CP cDNA | Combined | 33 | 3.4 | 2.9 | 0.2 | 0.8 |

| **Supplementary Table 4. Euclidian Distance of Colour Peak sediment 16S rRNA and 16S rRNA gene profiles of the Colour Peak sediment** | | | | |
| --- | --- | --- | --- | --- |
| **Euclidian Distance** | CP.cDNA | CP S1 | CP S2 | CP S3 |
| CP.cDNA | 0 | 14295 | 13150 | 13862 |
| CP S1 | 14295 | 0 | 2702 | 2522 |
| CP S2 | 13150 | 2702 | 0 | 2516 |
| CP S3 | 13862 | 2522 | 2516 | 0 |

| **Supplementary Table 5. Dice measure of Colour Peak sediment 16S rRNA and 16S rRNA gene profiles of the Colour Peak sediment** | | | | |
| --- | --- | --- | --- | --- |
| **Dice Dice** | CP.cDNA | CP S1 | CP S2 | CP S3 |
| CP.cDNA | 0 | 0.97 | 0.97 | 0.97 |
| CP S1 | 0.97 | 0 | 0.84 | 0.87 |
| CP S2 | 0.97 | 0.84 | 0 | 0.86 |
| CP S3 | 0.97 | 0.87 | 0.86 | 0 |

| **Supplementary Table 6. Chebyshev Distance of Colour Peak sediment 16S rRNA and 16S rRNA gene profiles of the Colour Peak sediment** | | | | |
| --- | --- | --- | --- | --- |
| **Chebyshev Distance** | CP.cDNA | CP S1 | CP S2 | CP S3 |
| CP.cDNA | 0 | 9178 | 7892 | 8665 |
| CP S1 | 9178 | 0 | 1286 | 658 |
| CP S2 | 7892 | 1286 | 0 | 773 |
| CP S3 | 8665 | 658 | 773 | 0 |

| **Supplementary Table 7. Details of microbes isolated from Colour Peak sediment** | | | |
| --- | --- | --- | --- |
| **Isolate** | **Closest match on SILVA** | **Environment of isolation of highest match** | **Media of isolation** |
| CP1 | *Halomonas neptunia* | Pacific hydrothermal vents ^121^ | DSMZ 63 |
| CP2 | *Halomonas taeanensis* | Saltern ^122^ | DSMZ 63 |
| CP3 | *Halomonas taeanensis* |  | LB |
| CP4 | *Psychrobacter piscatorii* | Yumoto Fish plant ^123^ | R2A |
| CP5 | *Psychrobacter piscatorii* |  | LB |
| CP6 | *Marinobacter antarcticus* | Antarctic sediment ^82^ | Dilute ammonia nitrate mineral salts + lanthanum |
| CP11 | *Loktanella salsilacus* | Arctic mat ^124^ | R2A |
| CP13 | *Loktanella salsilacus* |  | LB |
| CP14 | *Salegentibacter* | North Sea ^125^ | 0.3 % agarose DSMZ 63 |
| CP15 | *Sphingopyxis* *chilensis* | Phenol polluted river^126^ | DSMZ 63 |
| CP16 | *Sporosarcina* | Antarctic brine pockets ^127^ | SFM |
| CP17 | *Variovorax paradoxus* | Soil^128^ | DSMZ 63 |
| CP18 | *Variovorax paradoxus* |  | 0.3 % agarose DSMZ 63 |
| CP19 | *Acidovorax facilis* | Freshwater ^129^ | SFM |
| CP20 | *Nevskia ramosa* | Freshwater ^130^ | SFM |

| \| **Supplementary Table 8. Details of media used for enrichment and isolation of organisms from the Colour Peak sediment** \| \| \| \| \| \| \| \| \| --- \| --- \| --- \| --- \| --- \| --- \| --- \| --- \| \| **Media** \| **N Source** \| **C source** \| **Salinity (NaCl)** \| **Temperature (°C)** \| **Target** \| **Oxygen** \| **Reference** \| \| Methyl–reducing methanogen \| 1 mM ammonium \| 2 mM trimethylamine, acetate, formate \| 2M \| 4 \| Methanogens \| Anaerobic \| Sorokin *et al.*, 2017^131^ \| \| Methyl–reducing methanogen \| 1 mM ammonium \| 2 mM trimethylamine, acetate, formate \| 2M \| 22 \| Methanogens \| Anaerobic \| Sorokin *et al.*, 2017^131^ \| \| Marine SRB \| 1 mM ammonium + YE \| Yeast extract, peptone \| 2M \| 4 \| SRB \| Anaerobic \|  \| \| R2A \| YE + Peptone \| Yeast extract, peptone \| 2M \| 15 \| Heterotrophs \| Aerobic \| Reasoner and Geldreich^132^ \| \| Lysogeny Broth \| YE + Peptone \| Yeast extract, peptone \| 2M \| 15 \| Heterotrophs \| Aerobic \| Bertani 1951^133^ \| \| Dilute ammonia nitrate mineral salts + lanthanum \| 1 mM ammonium and nitrate \| 2 mM acetate \| 2M \| 15 \| Heterotrophs \| Aerobic \| Macey *et al.*, 2018^134^ \| \| DSMZ 63 \| 1 mM ammonium \| None \| 2M \| 22 \| Autotrophs \| Aerobic \| Sievert *et al.*, 2000^135^ \| \| DSMZ 63 \| 1 mM ammonium \| None \| 4M \| 22 \| Autotrophs \| Aerobic \| Sievert *et al.*, 2000^135^ \| \| DSMZ 63 \| 1 mM ammonium \| 2 mM methanol \| 2M \| 22 \| Methylotrophs \| Aerobic \| Sievert *et al.*, 2000^135^ \| \| 0.3 % agarose DSMZ 63 \| 1 mM ammonium and nitrate \| None \| 2M \| 15 \| Autotrophs \| Microaerophillic \| Sievert *et al.*, 2000^135^ \| \| 0.3 % agarose DSMZ 63 \| 1 mM ammonium and nitrate \| None \| 4M \| 15 \| Autotrophs \| Microaerophillic \| Sievert *et al.*, 2000^135^ \| \| Mannitol Soya Flour \| Soya flour \| Soya flour \| 1M \| 22 \| Heterotrophs \| Aerobic \| Hobbs, G. et al., 1989^136^ \| | | | | | | | |
| --- | --- | --- | --- | --- | --- | --- | --- | --- | --- | --- | --- | --- | --- | --- | --- | --- | --- | --- | --- | --- | --- | --- | --- | --- | --- | --- | --- | --- | --- | --- | --- | --- | --- | --- | --- | --- | --- | --- | --- | --- | --- | --- | --- | --- | --- | --- | --- | --- | --- | --- | --- | --- | --- | --- | --- | --- | --- | --- | --- | --- | --- | --- | --- | --- | --- | --- | --- | --- | --- | --- | --- | --- | --- | --- | --- | --- | --- | --- | --- | --- | --- | --- | --- | --- | --- | --- | --- | --- | --- | --- | --- | --- | --- | --- | --- | --- | --- | --- | --- | --- | --- | --- | --- | --- | --- | --- | --- | --- | --- | --- | --- | --- | --- | --- | --- | --- | --- | --- | --- |
|  |  |  |  |  |  |  |  |


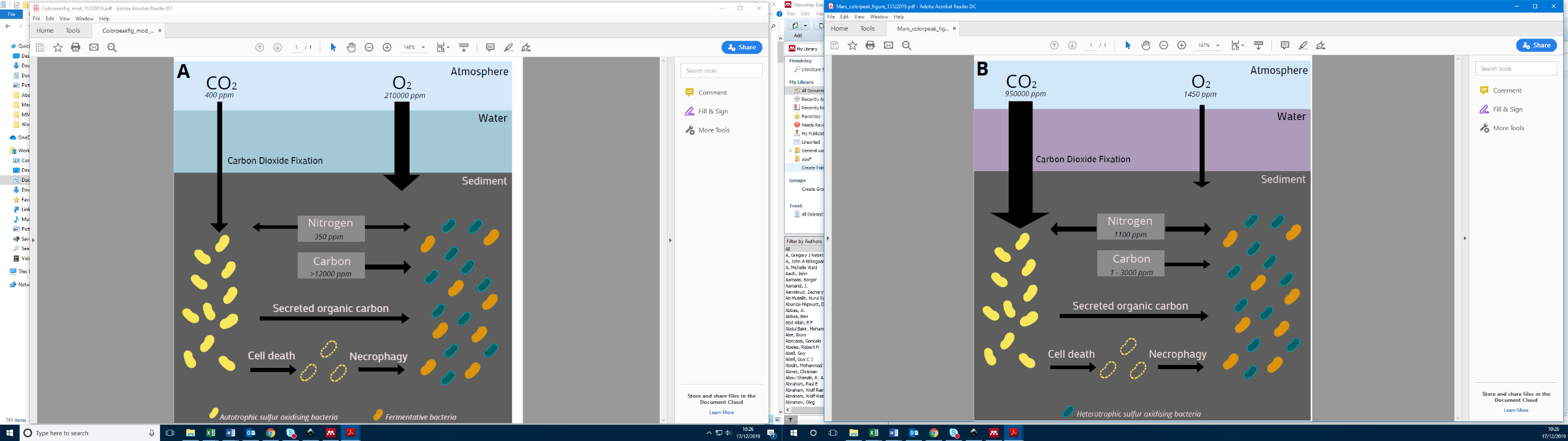

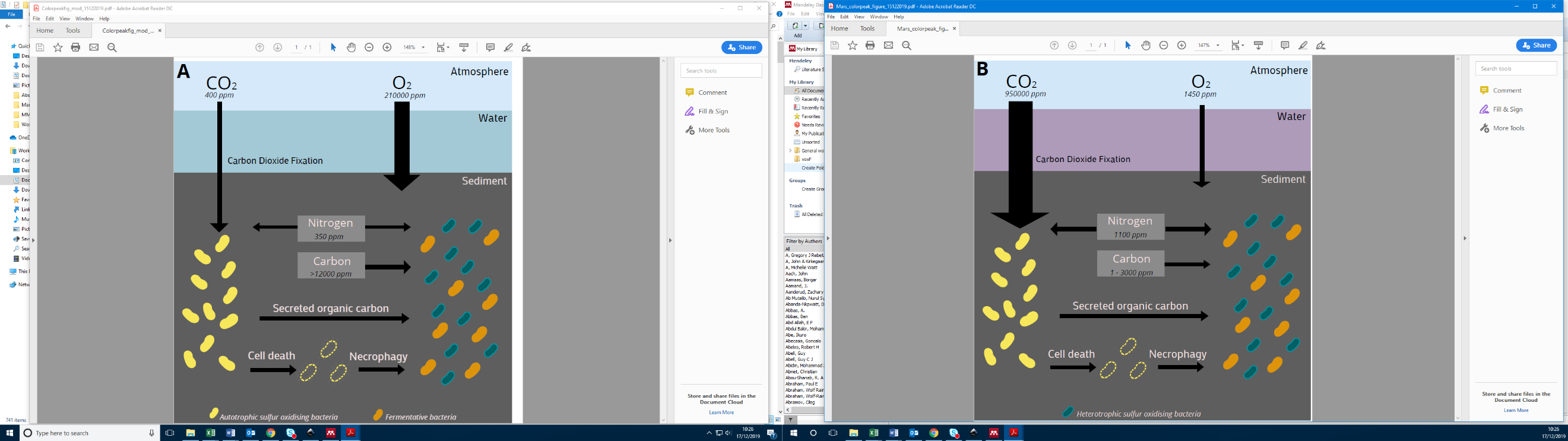


**Supplementary Figure 1. Proposed carbon and nitrogen cycles within the Colour Peak sediment and predicted aqueous environments present on the surface of Mars during the Noachian–Hesperian transition. Proposed community dynamics and carbon and nitrogen cycling based on the 16S rRNA community profile produced from RNA extracted from A) the Colour Peak sediment and values measured or estimated from previous studies characterising the Colour Peak sediment**^45^ **or B) the surface of Mars**^25,26^**.**
